# Quantitative analysis of *Nipah virus *proteins released as virus-like particles reveals central role for the matrix protein

**DOI:** 10.1186/1743-422X-4-1

**Published:** 2007-01-04

**Authors:** Jared R Patch, Gary Crameri, Lin-Fa Wang, Bryan T Eaton, Christopher C Broder

**Affiliations:** 1 Department of Microbiology and Immunology, Uniformed Services University, Bethesda, Maryland 20814, USA; 2 CSIRO Livestock Industries, Australian Animal Health Laboratory, Geelong, Victoria 3220, Australia

## Abstract

**Background:**

*Nipah virus *(NiV) is an emerging paramyxovirus distinguished by its ability to cause fatal disease in both animal and human hosts. Together with Hendra virus (HeV), they comprise the genus *Henipavirus *in the *Paramyxoviridae *family. NiV and HeV are also restricted to Biosafety Level-4 containment and this has hampered progress towards examining details of their replication and morphogenesis. Here, we have established recombinant expression systems to study NiV particle assembly and budding through the formation of virus-like particles (VLPs).

**Results:**

When expressed by recombinant Modified Vaccinia virus Ankara (rMVA) or plasmid transfection, individual NiV matrix (M), fusion (F) and attachment (G) proteins were all released into culture supernatants in a membrane-associated state as determined by sucrose density gradient flotation and immunoprecipitation. However, co-expression of F and G along with M revealed a shift in their distribution across the gradient, indicating association with M in VLPs. Protein release was also altered depending on the context of viral proteins being expressed, with F, G and nucleocapsid (N) protein reducing M release, and N release dependent on the co-expression of M. Immunoelectron microscopy and density analysis revealed VLPs that were similar to authentic virus. Differences in the budding dynamics of NiV proteins were also noted between rMVA and plasmid based strategies, suggesting that over-expression by poxvirus may not be appropriate for studying the details of recombinant virus particle assembly and release.

**Conclusion:**

Taken together, the results indicate that NiV M, F, and G each possess some ability to bud from expressing cells, and that co-expression of these viral proteins results in a more organized budding process with M playing a central role. These findings will aid our understanding of paramyxovirus particle assembly in general and could help facilitate the development of a novel vaccine approach for henipaviruses.

## Background

*Nipah virus *(NiV) is a newly recognized, emerging paramyxovirus capable of causing lethal infections in a number of mammalian species including humans [1]. Together, with Hendra virus (HeV), they are members of the recently created *Henipavirus *genus within the *Paramyxoviridae *family [2]. The type species HeV [3] appeared first in eastern Australia in 1994 and was transmitted to humans from infected horses (reviewed [4]). NiV was identified later during an outbreak of severe encephalitis in Malaysia and Singapore that began in 1998 and continued into 1999 and was primarily transmitted to humans from infected pigs, although several additional animal species were also noted to be infected (reviewed [5]). Subsequent outbreaks of NiV in Bangladesh [6-10] and India [11] have been smaller in scope but associated with higher mortality and some human-to-human transmission [8]. Their broad species tropism coupled with their highly pathogenic characteristics has distinguished the henipaviruses from all other paramyxoviruses (reviewed in [12]).

The natural hosts of HeV and NiV appear to be several species of flying foxes, bats in the genus *Pteropus *[13]. Evidence of henipavirus infection of bats has been obtained in Australia, Malaysia, Cambodia, and Thailand [14-17] and virus has been isolated from bat urine and partially eaten fruit [16,18]. Because of their availability from natural sources and relative ease of propagation and dissemination, NiV and HeV have been classified as priority pathogens by the Centers for Disease Control and Prevention (CDC) and the National Institute of Allergy and Infectious Diseases (NIAID). There are currently no approved vaccines or effective therapeutics for the prevention or treatment of NiV or HeV infection.

Like other paramyxoviruses, NiV and HeV are enveloped with single-stranded negative-sense RNA genomes that replicate in the cytoplasm [1,19]. Members of this family include several well-known viruses such as measles virus (MeV), Sendai virus (SeV), human parainfluenza viruses (hPIV) types 1–4, simian virus 5 (SV5), Newcastle disease virus (NDV), mumps virus, and respiratory syncytial virus [19]. The genome encodes six principal viral proteins: nucleocapsid (N) protein, phosphoprotein (P), matrix (M), the fusion (F) and attachment (H, HN, G) envelope glycoproteins, along with the viral RNA-dependent RNA polymerase (L). Additional viral proteins include V, C, and others that vary according to species [19]. Among the paramyxoviruses, comparatively little is known about the cell biology of the henipaviruses, but there have been several significant advances made in recent years through the analysis of the structure and function of several henipavirus proteins expressed from cloned genes, particularly the polycistronic P gene which encodes four proteins: P, V, C and W that have been shown to modulate virulence by abrogatingthe cellular interferon response [20-23]. Other studies on the F and G envelope glycoproteins, which together determine host range and cellular tropism [24,25], have identified EphrinB2 as a key cellular receptor for both NiV and HeV [26,27], and have also revealed a unique F precursor cleavage and maturation process [28-30]. However, an examination of henipavirus particle assembly and roles the various viral proteins may play in that biological process has not been described.

The assembly and morphogenesis of progeny virions requires that viral proteins, including the envelope glycoproteins and ribonucleoprotein (RNP) complex, associate at the plasma membrane for inclusion into budding virions. This association is thought to be mediated by the M protein; however the details of this process are poorly understood and seem to vary among viral species [31]. Recombinant MeV [32], SeV [33] and rabies virus [34] that lack M are impaired in budding ability but remain infectious as demonstrated by increased cell-cell fusion. Recombinant expression of the M protein of SeV [35,36], hPIV-1 [37], or NDV [38], in the absence of other viral proteins, leads to budding of virus-like particles (VLPs). Similar results have been observed for vesicular stomatitis virus (VSV) [39-41] and Ebola virus (EBOV) [42-46]. In addition, certain envelope glycoproteins also appear to have intrinsic budding activity, as has been shown for SeV F [35,36], the G protein of rabies virus (RV) and VSV [47,48], and the envelope glycoprotein (Gp) of Ebola virus [44,45,49]. In contrast, SV5 requires expression of M along with N and at least one of its envelope glycoproteins in order for efficient budding to occur [50].

NiV culture is restricted to BSL-4 containment and this imparts significant limitations on experimentation aimed at exploring the cell biology of the virus. To circumvent this, we used recombinant gene expression systems, both plasmid transfection-based and recombinant Modified Vaccinia virus Anakara (MVA), to safely study the viral proteins individually and together through the generation of VLPs in cell culture. Both vaccinia virus and MVA have been used in reverse genetics systems to generate negative-sense RNA viruses, including paramyxoviruses, from cDNA [51]. Vaccinia virus has also been employed in budding assays for both rhabdoviruses and filoviruses [39,41,42,49], however certain features of MVA suggested it may be a better platform for such assays. MVA is an attenuated deletion mutant of Vaccinia virus that cannot replicate in most mammalian cells [52]. The block in replication occurs during viral assembly, which allows for a high level of gene expression without progeny virus production and with less cytopathic effect [53]. Here we describe the generation and characterization of NiV VLPs. Our results demonstrate that NiV M possesses intrinsic budding activity and can facilitate the inclusion of other viral proteins into VLPs. Both the F and G envelope glycoproteins could also be independently released from expressing cells in association with membrane, however co-expression of these viral proteins resulted in a reduction of the level of M budding perhaps by virtue of a more organized assembly process with M playing a central role. Sucrose density gradient analysis and immunoelectron microscopy revealed particles consistent in density and size with authentic NiV. These findings will aid our understanding of paramyxovirus particle assembly in general and could help facilitate the development of novel vaccine approaches for henipaviruses.

## Results

### MVA expression of NiV proteins

**Figure 1:**
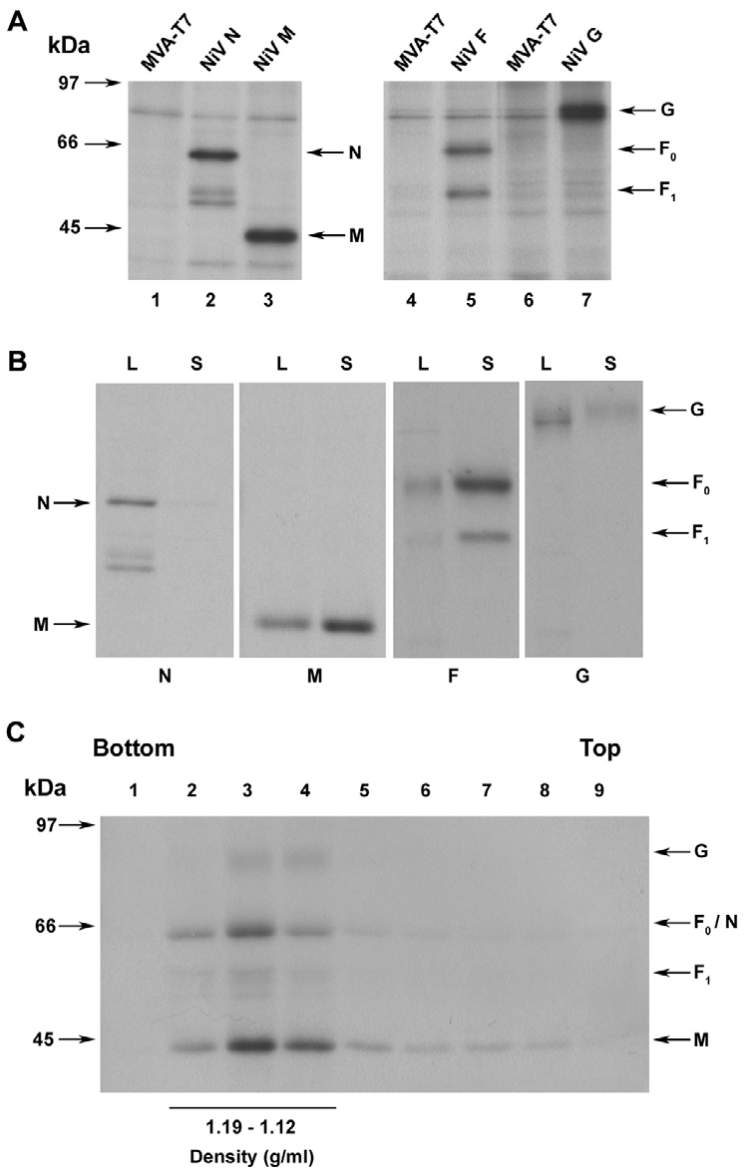
MVA expression of NiV proteins results in protein release. (A) Vero cells were infected with rMVAs expressing NiV N, M, F, or G, along with MVAGKT7 and then metabolically labeled. Lysates were immunoprecipitated with either rabbit anti-NiV polyclonal serum (N, M, G) or rabbit anti-F polyclonal serum. (B) Vero cells were infected with rMVAs and MVAGKT7 and metabolically labeled. Released protein was purified by centrifugation through a 10% sucrose cushion followed by flotation in a sucrose gradient. Proteins derived from cell lysates (L) or culture supernatants (S) were immunoprecipitated and analyzed as described above and in Methods. Lysate bands represent 1/20 of total lysate. (C) Supernatant from radiolabeled cells expressing N, M, F, and G was clarified and released protein was loaded onto a 5–45% sucrose gradient followed by centrifugation for 16 h as described in Methods. Fractions were obtained and proteins were analyzed by immunoprecipitation. A portion of each fraction was used to determine density.

We first sought to develop a NiV VLP expression system using the MVA poxvirus as a means of gene delivery and expression. Here, the NiV N, M, F and G ORFs were sub-cloned into the pMC03-based vector [54] in which the vaccinia virus early-late promoter was replaced with the bacteriophage T7 promoter. These constructs were then used to create the various rMVAs containing the individual NiV genes under the control of the T7 promoter. To test for NiV protein expression, Vero cells were infected with individual rMVAs expressing N, M, F, or G, along with MVAGKT7 encoding the T7 RNA polymerase. Infected cells were metabolically labeled overnight. Cell lysates were prepared and the NiV proteins were immunoprecipitated with NiV-specific polyclonal rabbit serum or rabbit anti-F polyclonal serum (Fig. 1). Immunoprecipitated proteins revealed bands corresponding to NiV N and M (Fig. 1A, lanes 2 and 3) which migrated at the expected apparent molecular weights of ~58 kDa (N) and ~42 kDa (M) respectively [55,56]. The NiV F_0 _(~61 kDa), F_1 _(~49 kDa), and G (~74 kDa) shown in Fig 1A (lanes 5 and 7), were found to be consistent with patterns reported previously [25].

**Figure 2:**
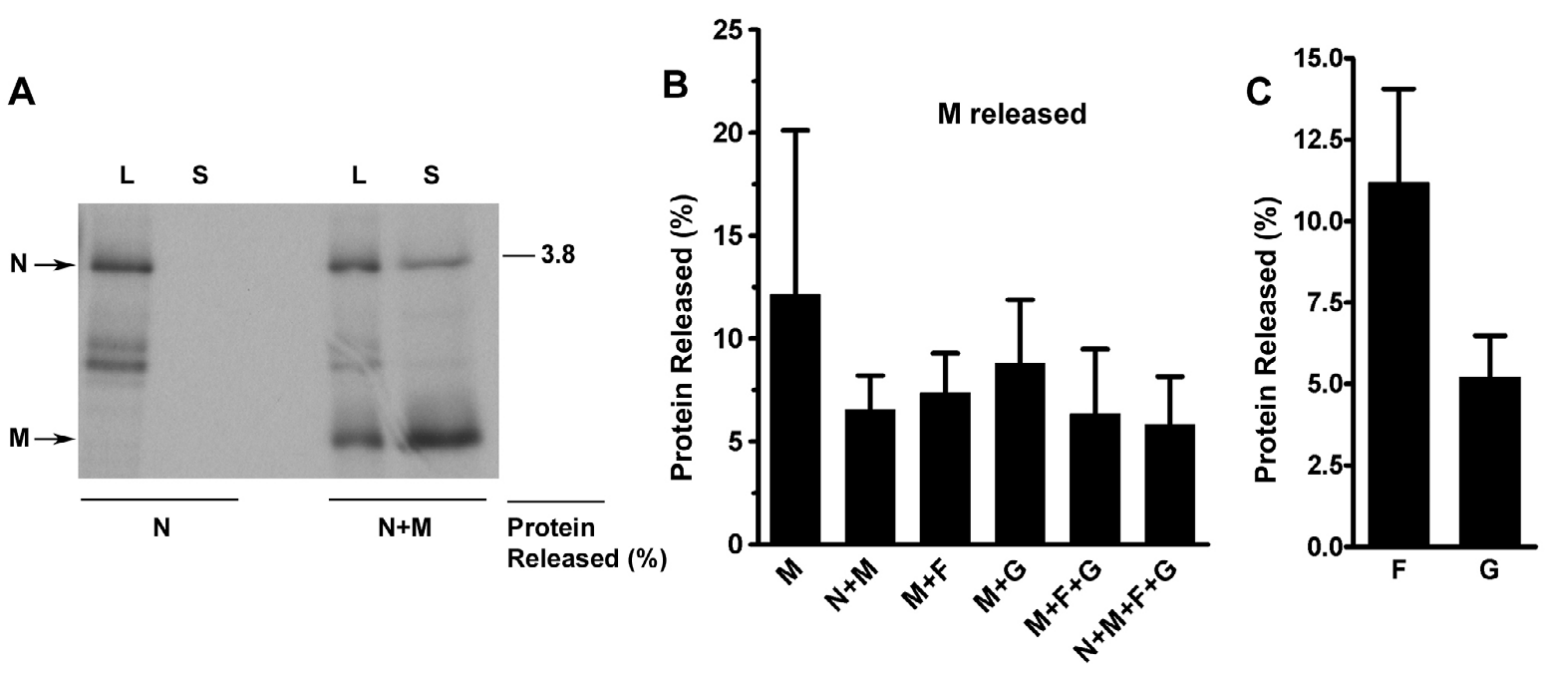
Quantitated release of MVA-expressed NiV proteins. Cells were infected with rMVAs in order to express NiV proteins alone or in combination. Cells and supernatants were treated as in Fig. 1B. Membrane-associated protein release of (A) N, (B) M and (C) F and G was quantitated by densitometry. A representative experiment is shown for A, and the mean and standard deviation of three or more experiments are shown in B and C.

In subsequent experiments to evaluate whether expression of NiV proteins can lead to VLP formation, cells were infected with rMVAs and metabolically labeled for 44 h followed by collection of both the cells and culture supernatant. Vesicles in the culture supernatant were pelleted by centrifugation through a 10% sucrose cushion and then floated by centrifugation in a discontinuous sucrose gradient as described in the Methods. Membrane-associated proteins were collected from the top of the gradient and detected by immunoprecipitation and separation by SDS-PAGE followed by autoradiography. While little membrane-associated N was detected in culture supernatants, individual expression of M, F, and G resulted in detectable membrane-associated protein release (Fig. 1B). To characterize the protein release as genuine VLPs, the culture supernatant of metabolically labeled cells expressing N, M, F, and G together was layered onto a 5–45% sucrose gradient, which was then centrifuged to allow membrane-associated proteins to migrate to their buoyant density. Fractions of the gradient were then removed with a portion of each fraction set aside for sucrose density determination. The proteins in the remaining portion of the fractions were then detected by immunoprecipitation followed by SDS-PAGE and autoradiography analysis. NiV proteins were found predominantly in fractions 2 through 4, which corresponded to a density range of 1.12–1.19 g/ml (Fig. 1C). This density range was consistent with the density reported for SeV and NDV VLPs [35,36,38], as well as authentic NiV (see below). To examine the contribution of each viral protein to overall protein release, NiV proteins were expressed in different combinations and their release was quantified. A release of N was not detected when expressed alone, however co-expression of M with N resulted in release of both proteins into the supernatant (Fig. 2A). Quantified expression of M with other viral proteins resulted in a reduction in the mean M released (~12%) compared to its expression alone; however this reduction did not reach statistical significance among repeated experiments (Fig. 2B). We also confirmed that expression of multiple proteins simultaneously did not reduce the overall N, M, F, or G expression levels (data not shown). Quantitation of envelope glycoprotein release revealed that approximately 11% of F and 5% of G was released when expressed alone (Fig. 2C).

### NiV protein release and VLP production is not dependent on MVA infection

Although less cytopathic than its wild-type parent, MVA retains the ability to block host protein synthesis and otherwise interfere with normal cellular metabolism [57]. In order to determine whether the protein release we observed was an accurate reflection of NiV biology rather than a potential artifact resulting from poxvirus infection, we employed a transfection plasmid-based expression system using the pCAGGS eukaryotic expression vector, which has also been used in other paramyxovirus VLP assays [35-38,50]. The NiV N, M, F, and G genes were sub-cloned into the pCAGGS vector and expression was verified in separate experiments by immunoprecipitation (data not shown).

**Figure 3:**
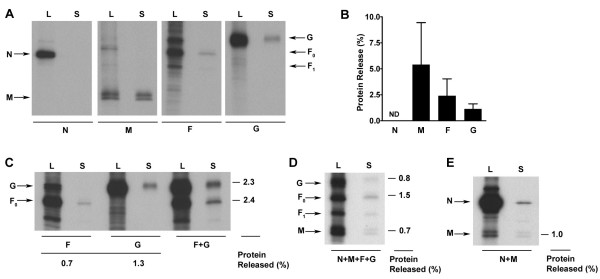
NiV proteins are released in the absence of MVA. 293T cells were transfected with pCAGGS constructs containing NiV genes. (A) Protein released into the supernatant was purified by gradient centrifugation as described in Methods. Proteins were immunoprecipitated with MAbs F45G6 (anti-N), F45G5 (anti-M) or rabbit polyclonal anti-serum against NiV F or HeV G. (B) Protein released as in (A) was quantitated by densitometry. The mean and standard deviation of three or more experiments are shown. ND = not detectable. Release of protein from cells expressing combinations of F and G (C) N, M, F, and G (D) or N and M (E) are shown along with the percent protein released. N was not immunoprecipitated in D to allow visualization of F_0_. Quantitation for C was performed on a lighter exposure. Proteins derived from cell lysates (L) or culture supernatants (S) are indicated. Lysate bands represent 1/6 of total lysate. Representative experiments are shown.

To determine whether the various NiV proteins produced by this method were also released from expressing cells, cultures were transfected with individual plasmid constructs for 24 h, followed by metabolic labeling for another 20 h. Cells and culture supernatants were harvested and processed as described above and in the Methods. We again observed membrane-associated release of M, F and G, but no detectable release of N as predicted (Fig. 3A). Immunoprecipitation analysis of M expressed by plasmid-transfected cells consistently revealed a doublet of bands that were both released into the culture supernatant (Fig. 3A). When M was expressed by MVA, the doublet was usually only revealed by Western Blot (data not shown). We have been unable to determine the nature of the doublet, but it could reflect a post-translational modification of M. Although NiV M does contain a second potential AUG start codon 36 nt downstream of the first start codon [58], this does not account for the doublet appearance because a truncation mutant that begins at the second start codon, as well as HeV M, which lacks the second AUG codon, also appear as doublets (data not shown). Quantification of protein release revealed an overall reduction in protein release compared to that seen in the MVA system (Fig. 3B, compare with Fig. 2). Several studies have provided evidence for a physical interaction between paramyxovirus attachment and fusion proteins [59-61]. For NiV F and G this interaction is apparent from their ability to be co-precipitated without cross-linking (Bossart and Broder, unpublished data). We therefore sought to determine the effect of F and G co-expression on their release. Expression of F and G together resulted in greater release of both proteins together in comparison to expression of each protein individually (Fig. 3C). This observed increase of their release did not appear to be a result of increased protein expression or cell-surface expression (data not shown). We also noted that when N, M, F and G were all co-expressed using equivalent amounts of transfected plasmids, there was an overall reduction in protein release from cells (data not shown). Indeed, in certain other recombinant VLP expression systems, the amounts of transfected plasmids have been adjusted in order to more accurately reflect or achieve cellular expression levels of the individual viral proteins produced using infectious virus [36,38,50]. In an attempt to increase protein release in our system, we performed similar experimental variations in the amounts of tranfected plasmids and estimated envelope glycoprotein expression in authentic NiV infection as one third of M expression and adjusted plasmid ratios accordingly [55,62,63], and we further reduced N expression to 50 ng per well. However, even when the adjusted plasmid ratio was used, overall protein release remained low (Fig. 3D). We verified that this was not due to a reduction of protein expression due to multiple gene expression (data not shown), so we interpret this as indicative of a more organized assembly and budding process. In addition, the co-expression of N and M resulted in the release of N but with modest release of both proteins (Fig. 3E). This was in agreement with our previous results obtained using the MVA expression system, which demonstrated that M facilitated the release of N. However, in contrast to the MVA expression system, here we observed that N reduced overall M release. (Fig. 3E, compare with Fig. 2A and 2B).

### Specific M release and kinetic analysis

**Figure 4:**
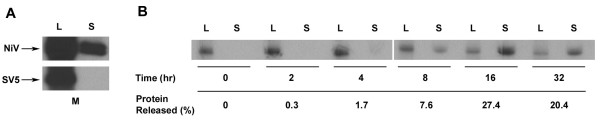
Comparison of NiV M release with SV5 M and kinetics of NiV M release. (A) Culture supernatants were analyzed for release of either NiV or SV5 M. Released protein was treated as described in Methods and proteins were immunoprecipitated using either rabbit anti-NiV polyclonal serum or MAb M-h (anti-SV5 M). Image shown is over-exposed to show contrast. (B) Cells expressing NiV M were starved for 45 min, ^35^S-pulsed for 15 min, then chased with DMEM-10 for times indicated. Released protein was purified and protein derived from lysate or supernatant was immunoprecipitated with MAb F45G5. Percent protein release was quantitated by densitometry. Proteins derived from cell lysates (L) or culture supernatants (S) are indicated. Lysate bands represent 1/6 of total lysate.

In order to ensure that NiV M release was accomplished by a mechanism specific to itself, release of NiV M was again examined but in parallel with SV5 M, which requires co-expression of N and one of the envelope glycoproteins in order to form VLPs that are released into the culture supernatant [50]. Here the analysis of culture supernatants showed release of NiV M but no detectable release of SV5 M (Fig. 4A), suggesting that the observed release of NiV M was specific to its biological properties and not due to a non-specific mechanism such as cell lysis. In this respect, NiV M was similar to the M proteins of SeV, NDV, and hPIV1, all of which have been reported to form VLPs in the absence of other viral proteins [35-38].

We also wanted to explore the kinetics of M expression and its eventual release from cells. For this analysis, plasmid-transfected cells were starved for 45 min, ^35^S-pulsed for 15 min, and then chased for varying lengths of time up to 32 h. At each time point cells and supernatants were harvested. Vesicles released into the supernatant were purified by centrifugation through a 10% sucrose cushion followed by sucrose gradient flotation. M derived from supernatants or cell lysates was immunoprecipitated with a monoclonal antibody and visualized by SDS-PAGE and autoradiography. Percent M release was quantitated by densitometry. The presence of membrane-associated protein in the supernatant was barely detectable at 2 h with 0.3% release, but then readily detected at 4 h with 1.7% release (Fig. 4B). Maximum M release of 27.4% was observed by 16 h.

### Electron microscopy analysis of VLPs

**Figure 5:**
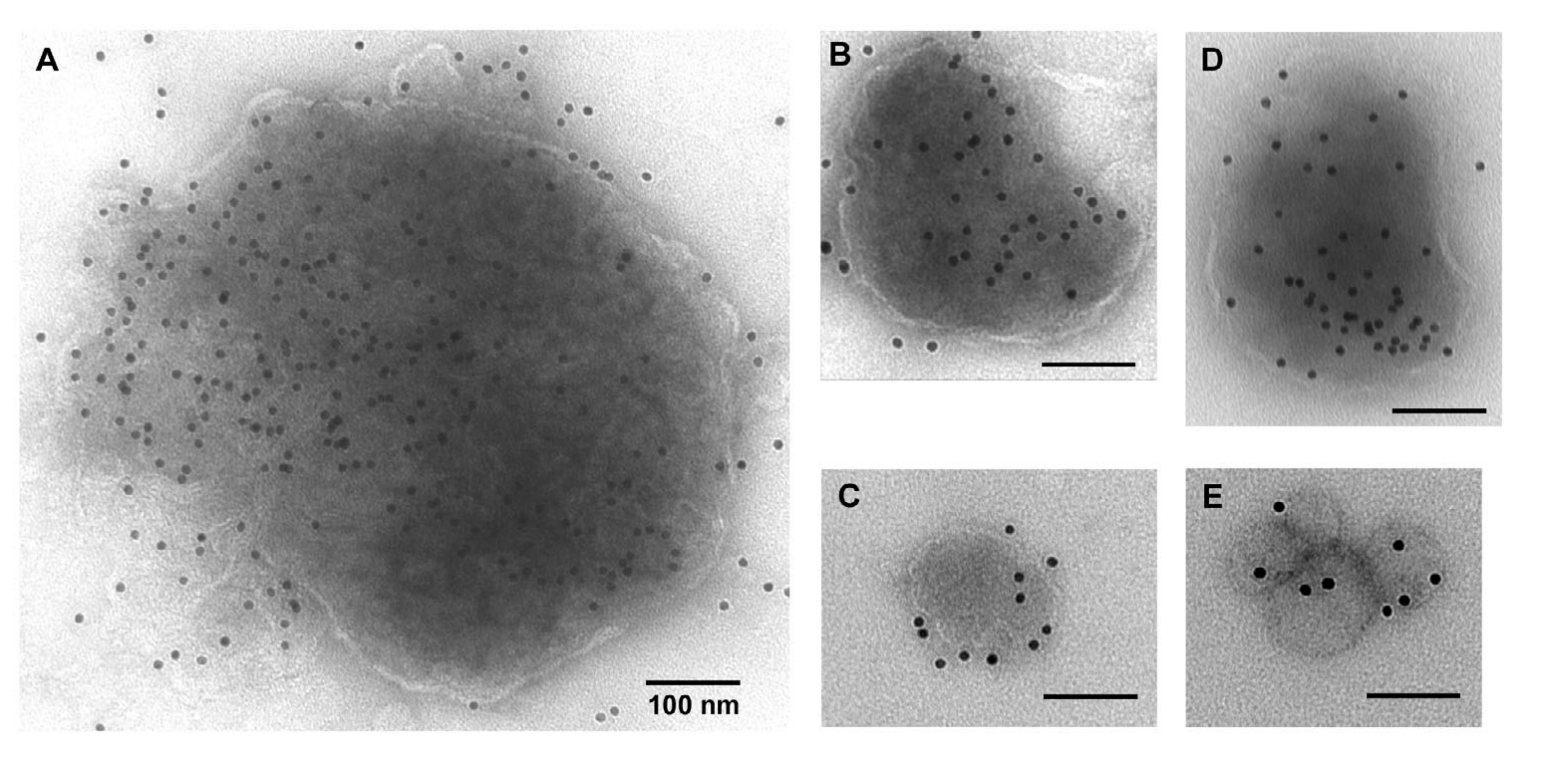
Immunoelectron microscopy shows particles consistent in size with authentic NiV virions. VLPs released from transfected 293T cells were purified by gradient centrifugation, labeled by immuno-gold, negatively stained, and viewed by electron microscopy as described in Methods. VLPs were derived from cells that expressed M (A to C) or N, M, F, and G (D and E). Immunolabeling against M was done using MAb F45G5 (A to D) and G was detected with mouse anti-HeV G polyclonal serum (E). Bars represent 100 nm. Specificity of primary and secondary antibodies was demonstrated by their failure to stain negative controls (not shown).

The biochemical analysis of the NiV proteins released from expressing cells in a membrane-associated manner suggested that VLPs were being generated. To analyze the released material visually, VLPs were prepared and isolated by sucrose gradient floatation and the resulting fractions were immunolabeled using antibodies against NiV M or HeV G followed by a secondary antibody conjugated to gold beads. After negative staining, the samples were examined by immunoelectron microscopy. Expression of NiV M alone resulted in the release of VLPs that contained M and varied in size from approximately 100 to 700 nm in diameter (Fig. 5A–C). Expression of NiV N, M, F, and G resulted in the release of VLPs with detectable M (Fig. 5D) or G (Fig. 5E) with a diameter of approximately 100 to 300 nm. The size of the VLPs observed is consistent with observations made on authentic NiV virions which have been reported with sizes ranging from 40 to 1900 nm [64,65].

### Interaction between proteins during VLP formation

**Figure 6:**
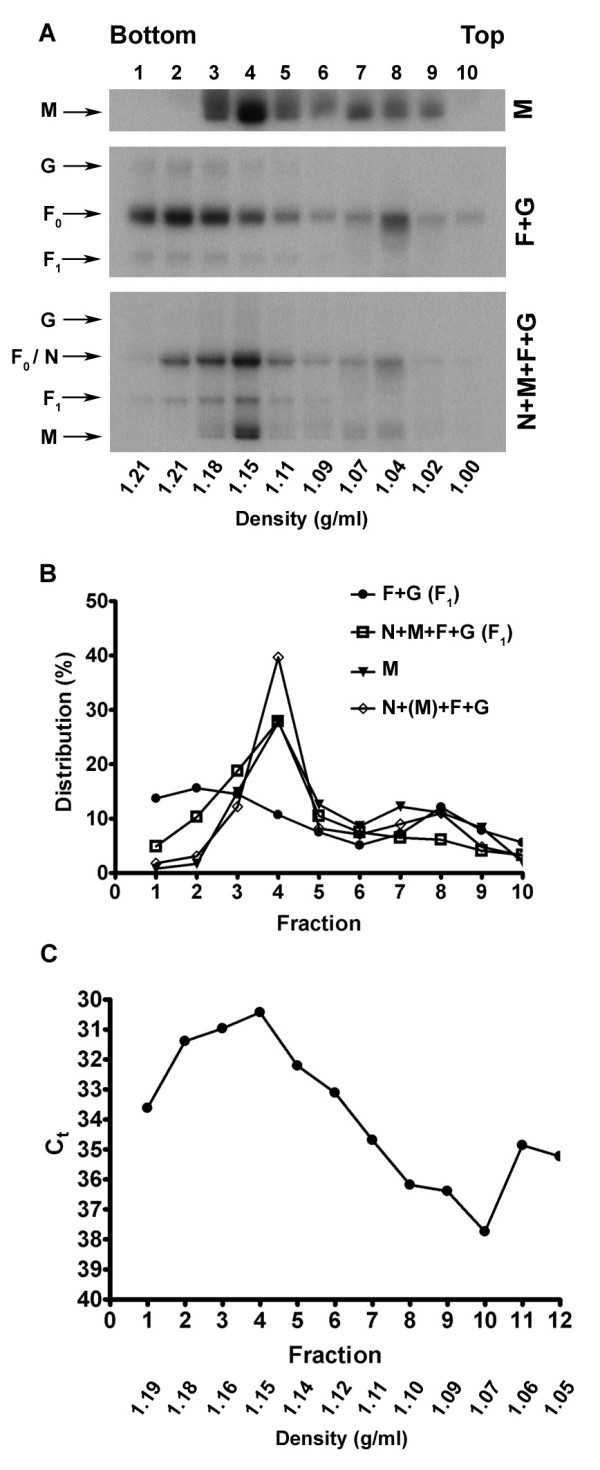
Density analysis of VLPs released from transfected cells expressing NiV proteins. (A) Clarified culture supernatants from cells expressing different combinations of NiV proteins were layered onto 5–45% sucrose gradients and centrifuged. Fractions were immunoprecipitated with MAbs F45G6 (ant-N), F45G5 (anti-M) or rabbit polyclonal anti-serum against NiV F or HeV G. A portion of each fraction was used to determine density. Densitometry analysis of the gels shown in (A) was used to determine the quantitative distribution of proteins (B). Where more than one protein was expressed, the protein analyzed by densitometry is indicated by parentheses. (C) Supernatant from cells infected with NiV was layered onto a 5–45% sucrose gradient and centrifuged. RNA was extracted from fractions and used for real-time PCR. The threshold cycle (Ct) value was used as a proxy for virus concentration.

In order to further investigate the interaction of F and G with M during VLP release we performed sucrose density gradient analyses to determine whether the buoyant density of released particles varied depending on the viral proteins present. M produced alone, F produced with G, or the combination of N, M, F and G were expressed in cells, metabolically labeled, and the culture supernatants were harvested and layered onto 5–45% continuous sucrose gradients. After centrifugation, the gradients were fractionated and analyzed by immunoprecipitation and SDS-PAGE. The M protein was again recovered predominantly in fractions 3 to 5, corresponding to a density range of 1.11–1.18 g/ml (Fig. 6A and 6B). We noted that the M protein could also be found in less dense fractions, especially when it was expressed in the absence of any other viral proteins. When F was co-expressed along with G, both proteins were recovered predominantly in fractions 1 to 3, which correspond to a density of 1.18–1.21 g/ml (Fig. 6A and 6B). However, the co-expression of N, M, F, and G led to a greater concentration of M in fraction 4 and, importantly, this also resulted in a shift of the fractionation profile of F and G distribution to fractions 3 to 5 (Fig. 6A and 6B). This density range was also consistent with the previous results obtained using rMVAs (Fig. 1C). Further, sucrose density gradient analysis of authentic infectious NiV revealed peak virus concentration at a density of 1.15 g/ml, which corresponded well with our VLP results (Fig. 6C). Taken together these findings suggest that although F and G can direct budding when produced alone, the co-expression of M facilitates F and G incorporation into VLPs with a density that is more consistent with that of an authentic virion.

## Discussion

The advent of reverse-genetics coupled with the *in vitro *techniques of VLP production has made it possible to examine the cell biology of viruses in increasing detail. However, certain highly pathogenic viruses such as the henipaviruses are restricted to BSL-4 containment, making such studies impractical, and in the absence of a henipavirus reverse-genetics system there are considerable obstacles in place to dissecting out the details of an individual protein's role(s) in particle formation. To circumvent some of these obstacles, we have established a VLP system to allow us to investigate certain details of NiV assembly and release. Our first attempts made use of a recombinant poxvirus platform using MVA because of its high efficiency in gene delivery and expression, and for its reduced level of cytopathic effect as compared to vaccinia virus. Further, poxviruses have been successfully employed in reverse-genetics systems and virus budding assays with success [39,41,42,49,51]. Using rMVAs to deliver NiV genes we observed membrane-associated release of M, F, and G. The ability of an M protein to be released from cells when expressed alone has also been observed for some other paramyxoviruses including SeV [35,36], hPIV-1 [37], and NDV [38], as well as viruses in other related virus families including Ebola [42-45] and VSV [39-41]. In addition, Ciancanelli and Basler have recently independently reported that expression of NiV M leads to VLP formation [66]. Release of F when expressed alone has been observed for SeV [35,36], and release of HN has been noted for NDV [38]. Ebola virus [44,45,49], VSV [47,48], and rabies virus [47,48] each contain a single envelope glycoprotein that can also direct budding of vesicles, thus our observations here that NiV F and G can be independently released is not unprecedented. In contrast to these other viruses, individual viral proteins expressed alone from SV5 are not released from cells, and particle formation requires the expression of M, N, and at least one envelope glycoprotein in order for any significant VLP release to occur [50].

Typically, viruses rescued using reverse-genetics techniques that employ vaccinia virus are subsequently purified from contaminating poxvirus and amplified by growth in cell culture. In such cases, the alteration of cellular metabolism by vaccinia virus is therefore of little concern because it is ultimately unimportant to the outcome. However, we were concerned that the effects of MVA infection might confound the interpretations of our VLP studies. Therefore, we employed a eukaryotic plasmid-based system as a means of gene expression in order to determine whether the results obtained using rMVAs faithfully reflected NiV biology.

When individual NiV proteins were produced in cells by transfection, M, F and G were each independently released into culture supernatants in a membrane-associated manner. In contrast, N was not detected in culture supernatants, as predicted, and these results were in agreement with those observations made using rMVAs. However, the percent of protein released was higher in the MVA system, which may reflect an effect of MVA on cellular metabolism or overall expression levels of the individual genes. When M was expressed in combination with various NiV proteins using rMVAs we observed little change in M release, irrespective of which other NiV proteins were present. Expression of M with N enabled greater release of membrane-associated N, which is consistent with the model that M and N interact, and that this interaction facilitates the incorporation of the genome into budding virions [31,37,67]. The dynamics of protein release were somewhat different when a plasmid-based transfection and expression system was used. Under these conditions, we found that co-expression of M with N, or M with N, F and G lead to a reduction in M release. We obtained a similar result when F and M were co-expressed (data not shown). We also found that F and G co-expression lead to greater release of both proteins than when either was expressed independently. Notably, in either the MVA or plasmid-based expression systems we predominantly detected F_0 _in both cell lysates and supernatants (Fig. 1B and Fig. 3A, C–D). In contrast, NiV virions appear to contain completely processed F [68]. The reason for this difference is not known, but we and others have consistently observed greater levels of F_0 _when recombinant expression systems are employed which could bias its incorporation into particles [25,28,69], or perhaps additional viral factors present during natural viral replication result in greater F processing or a biased incorporation of processed F.

The context-dependent variations in protein release and buoyant density suggest that the viral proteins interact either directly or indirectly to orchestrate the particle budding process. The differences observed in the protein release when a comparison is made between the transfected plasmid-based and rMVA systems are likely a result of the alterations of cellular metabolism brought about by MVA infection. Although VLPs can be produced using either system, our results suggest that the two methods have different applications. We believe the cell biology of NiV is more accurately reflected in the plasmid-based transfection system in the absence of poxvirus infection. Because of the efficiency of gene delivery and expression, and the greater percentage of viral protein released, the MVA system could be more useful when quantity of VLPs is of primary concern. VLPs have been reported to elicit a protective immune response against Ebola and Marburg viruses in animal models [70], and might serve as a more efficient method of NiV VLP production for immunization studies or perhaps as a potential livestock vaccine, which we are exploring.

To ensure that NiV M was not released into culture supernatants by a non-specific means such as cell lysis, we assayed SV5 M in parallel as a control. As expected, we detected the release of M for NiV but not for SV5. However, SV5 M budding could readily be detected when it was co-expressed with its HN, F, and N genes (data not shown). Pulse-chase analysis of NiV M expression revealed detectable release from expressing cells by 2 to 4 h with a maximal release of at least 27% by 16 h. This percentage of release is, of course, greater than that observed during standard budding assays, which were not performed as a pulse-chase. The percentages of protein release reported in our budding assays here, as well as for SeV [35,36] and SV5 [50] have also been reported as non-pulse-chased systems. As a result, any nascent protein produced in the cell is included in the overall calculation. This method is useful as a relative measure between groups, but probably underestimates the true budding efficiency. Here, pulse-chase not only reveals the timing of release but also likely gives a more accurate picture of the kinetics of M production and its ultimate destination, that is its release from producing cells.

Ultrastructural studies of NiV have revealed pleiomorphic virions that range from 80–1900 nm [64,65]. Here, when M was expressed alone or along with N, F and G, we observed VLPs containing the various proteins by immuno-gold labeling and electron microscopy. The VLPs ranged in size from approximately 100 to 700 nm, consistent with the range reported for actual virus. Using both the MVA and transfection systems we observed NiV proteins migrating as membrane vesicles at a density range of 1.11–1.19 g/ml. Authentic NiV migrated to a similar density range with peak detection at 1.15 g/ml. This range is consistent with the density of SeV and NDV VLPs as well as SeV virions [35,36,38]. Using a plasmid transfection system we further evaluated the release of M alone, and with co-expression of N, F, and G. Although a greater percentage of M was detected in additional fractions when expressed alone, it was predominately concentrated in fractions 3 to 5 when co-expressed along with N, F, and G. Particles containing F and G migrated to denser fractions when additional NiV proteins were absent, but the position of both proteins in the gradient shifted to fractions 3 to 5 when M was present, suggesting that M interacts with F and G either directly or indirectly during assembly to facilitate the incorporation the envelope glycoproteins into the particles. The reason for the greater density of particles containing only F and G is unknown, but it is likely a reflection of altered lipid or protein incorporation relative to particle size. Taken together, the density and ultrastructural characteristics of the membrane-associated NiV proteins reported here are suggestive of authentic VLP formation.

Ciancanelli and Basler recently observed NiV VLPs when M was expressed alone or with envelope glycoproteins [66], which is supportive of our data. Although they did not perform quantitative analysis in their study, M release was apparently unaffected by the presence of one or both of the envelope glycoproteins. As mentioned above, in our hands plasmid-based co-expression of M and F resulted in a distinct reduction in M release. This apparent discrepancy may be attributable to differences in the VLP purification techniques, especially in our inclusion of an additional particle flotation step. Another difference between the two studies is our inclusion of the N protein, which was found to also reduce M release and this was not examined in the Ciancanelli and Basler report. VLP formation has been evaluated for other paramyxoviruses including SeV, hPIV-1, SV5, and NDV [35-38,50]. Of these, only SV5, SeV, and NDV have been addressed with any quantitative assessment. Two independent studies of SeV VLP formation reported that M and F can each be released independently and that when M and F are expressed together that the percentage of each protein released is greater [35,36]. However the percent of protein released differed dramatically between the studies with Takimoto *et al*. [35], reporting approximately 50% of M released and Sugahara *et al*. [36], reporting 14.5% of M released. Using avian cells and a pulse-chase system, Pantua *et al*. reported that NDV M is both necessary and sufficient for VLP release, with solo expression of NDV M resulting in 90% release efficiency [38]. Whereas SV5 VLP release was most efficient (32% of M released) when N, M, F, and HN were expressed together [50], there was a decrease in M release when the equivalent SeV or NDV proteins were co-expressed [36,38], which is comparable to our results observed with NiV N, M, F and G.

Sugahara et al. [36] have also shown that expression of the C protein with N, M, F, and HN leads to an increase in VLP release by 2- to 3-fold. This increase was subsequently shown to be due to the interaction of C with AIP1/Alix, a cellular protein involved in multivesicular body formation; however an interaction between AIP1/Alix and measles C was not detected, suggesting that this mechanism of budding is not applicable to all paramyxoviruses [71]. Nevertheless, this raises the possibility that additional NiV proteins not evaluated here may increase VLP release, but further experiments will be required to determine whether this is the case. It is also important to recognize that because the various studies on several paramyxovirus VLPs each employed slightly differing methodologies in their analysis, the calculated percentage of released proteins among the various reports are not directly comparable. However, the qualitative differences seen between these viruses suggest that beneath the generalized model of paramyxovirus assembly and budding lie differences in the specific mechanisms employed, and these differences remain to be determined.

## Conclusion

Nipah Virus-like particles can be produced by recombinant gene expression. The matrix, fusion, and attachment proteins each possess some budding ability of their own; however, the matrix protein appears to play a central role in virus particle assembly and release from expressing cells. The system developed here along with the present findings will help facilitate studies on NiV morphogenesis in greater detail, and provide a platform for exploring the nature and role of potential host factors in the virus budding process. Importantly, the VLP system detailed here also allows for the examination of recombinant particle assembly and release outside high-level biological containment.

## Methods

### Cell lines

Vero cells, provided by Alison O'Brien (Uniformed Services University), and chicken embryo fibroblast cells (Charles River Laboratories, Inc, Wilmington, MA) were maintained in Eagle's minimal essential medium (Quality Biologicals, Gaithersburg, MD) supplemented with 10% cosmic calf serum (Hyclone, Logan, UT), 2 mM L-glutamine, and 100 units/ml penicillin and streptomycin (Quality Biologicals, Gaithersburg, MD) (EMEM-10). 293T cells were maintained in Dulbecco's modified Eagle's medium (Quality Biologicals, Gaithersburg, MD) supplemented as described above (DMEM-10). All cultures were maintained at 37°C in 7.5% CO_2_.

### Antibodies

The following antibodies were used in immunoprecipitations: Polyclonal rabbit antiserum against NiV F was obtained by immunization of rabbits with a synthetic peptide of the following sequence: CNTYSRLEDRRVRPTSSGDL, which corresponds to the cytoplasmic tail of NiV F. The peptide was conjugated to keyhole limpet hemocyanin (KLH) for immunization. Monoclonal antibodies (MAbs) F45G5 (anti-M) and F45G6 (anti-N) were kindly provided by Jody Berry and Hana Weingartl (National Centre for Foreign Animal Disease, Canadian Food Inspection Agency). MAb M-h (anti-SV5 M) was kindly provided by Robert Lamb (Northwestern University). Mouse antiserum specific for HeV G was provided by Andrew Hickey (Uniformed Services University). Polyclonal serum from a rabbit immunized with gamma-irradiated NiV and from a rabbit immunized with soluble HeV G were also used.

### Plasmid and recombinant MVAs

A system for recombinant gene expression using modified vaccinia virus Ankara (MVA) has been described [54]. pMC03ΔE/L, which was made by removing the vaccinia virus promoter from pMC03 by ligation of the vector after *Pst*I digestion, was provided by Katharine Bossart (CSIRO Livestock Industries, Australian Animal Health Laboratory). To introduce the bacteriophage T7 promoter into the vector, complementary oligonucleotides 5'-ggaaattaatacgactcactatagggagaccacaacggtttaaacggcgcgccgga (T7S) and 5'-gatctccggcgcgccgtttaaaccgttgtggtctccctatagtgagtcgtattaatttcctgca (T7AS) were mixed in equal molar amounts, heated to 65°C, then allowed to cool and ligated into the *Pst*I and *Bgl*II sites of pMC03ΔE/L to form pMC03T7.

The NiV N ORF was PCR amplified from pCP629 (NiV N gene in pFastBac HTc) using primers 5'-GTTTAAACCACCATGAGTGATATCTTTG (NIVNS) and 5'-GTTTAAACTCACACATCAGCTCTG (NIVNAS). The NiV M ORF was PCR amplified from pCP630 (NiV M gene in pFastBac HTa) using primers 5'-GTTTAAACCACCATGGAGCCGGACATC (NIVMS) and 5'-GTTTAAACTTAGCCCTTTAGAATTCTC (NIVMAS). The NiV F ORF was PCR amplified from pMC02 NiV F [25] using the primers 5'-GTTTAAACCACCATGGTAGTTATACTTGAC (NF2) and 5'-GGCGCGCCCTATGTCCCAATGTAGTAG (NFAS2). The NiV G ORF was PCR amplified from pMC02 NiV G [25] using the primers 5'-GTTTAAACCACCATGCCGGCAGAAAAC (NIVGS) and 5'-GTTTAAACTTATGTACATTGCTCTGG (NIVGAS). PCR was done using Accupol DNA polymerase (PGS Scientifics Corp., Gaithersburg, MD) with the following settings: 94°C for 5 min, then 25 cycles of 94°C for 1 min, 55°C for 2 min, then 72°C for 3 min. The resulting PCR products were sub-cloned into pCRII-Blunt-TOPO (Invitrogen, Carlsbad, CA). TOPO constructs were digested with *Pme*I or *Pme*I and *Asc*I, as appropriate, and inserted into *Pme*I or *Pme*I-*Asc*I sites in pMC03T7.

The pCAGGS/MCS eukaryotic expression vector has been described previously [72,73]. In order to introduce an *Asc*I site into pCAGGS, 128 pmol of the oligonucleotide 5'-TCGACGGCGCGCCG (CAG1) was heated to 65°C, allowed to cool, and then ligated into the *Xho*I site of pCAGGS/MCS to form pCAGGS-AscI. The ORFs for NiV N, M, and G, were digested from pMC03T7 as *Pme*I fragments and ligated into the pCAGGS *Sma*I site. The ORF for NiV F was digested from pMC03T7 as a *Pme*I-*Asc*I fragment and ligated into the pCAGGS-AscI *Sma*I-*Asc*I site. pCAGGS-SV5 M was kindly provided by Robert Lamb.

To create recombinant MVAs, chicken embryo fibroblasts (CEFs) were infected with wild-type MVA at a multiplicity of infection (MOI) of 0.1. At 2 h post-infection, the CEFs were transfected with 8 μg of the appropriate pMC03T7 construct using Profection Mammalian Transfection System – Calcium Phosphate (Promega, Madison, WI). At 4 hr post-transfection, cells were washed, given fresh EMEM-10, and then incubated for 3 days at 37°C. Cells were harvested by scraping, pelleted, and then resuspended in 0.5 ml EMEM-2.5 as crude recombinant virus. After 3 cycles of freezing and thawing, virus was diluted and CEF monolayers were infected overnight at 37°C. Monolayers were then overlaid with EMEM-10 containing 1% low-melting point agarose (Invitrogen, Gaithersburg, MD) and incubated for 2 days. A final overlay of EMEM-10 containing 1% low-melting point agarose and 0.2 mg/ml 5-bromo-4-chloro-3-indolyl-β-D-glucuronic acid (X-Gluc) (Clontech, Palo Alto, CA) was added to the monolayers and over the following 72 h cells that showed blue staining were picked, resuspended in 0.5 ml EMEM-2.5, and used for repeated positive selection. After 5 or more rounds of purifying positive selection, recombinant MVAs were amplified in CEFs to make crude stocks. Recombinant MVA expressing the bacteriophage T7 RNA polymerase (MVAGKT7) was provided by Gerald R. Kovacs (National Institutes of Health, Bethesda, MD).

### Transfection, MVA infection, and metabolic labeling

293T cells in 6-cm wells were transfected in duplicate with pCAGGS constructs using FuGene 6 transfection reagent (Roche, Indianapolis, IN) according to the manufacturer's instructions. Unless otherwise specified, 1 μg of each plasmid was used per well. Empty vector was used to make the total DNA amount 4 μg/well. Vero cells were infected with recombinant MVAs at an MOI of 3–5 in a minimal volume of EMEM containing 2.5% serum. At 24 h post transfection or 6 h post infection, cells were overlaid with methionine-cysteine-free minimal essential medium (MEM) (Invitrogen, Gaithersburg, MD) containing 2.5% dialyzed fetal calf serum (Invitrogen, Gaithersburg, MD) and 100 μCi/ml ^35^S-cys/met Redivue Promix (Amersham Pharmacia Biotech, Piscataway, NJ), and incubated at 37°C for transfected cells, or 31°C for infected cells. For pulse-chase labeling, cells were washed then starved in methionine-cysteine-free MEM for 45 min followed by metabolic labeling as, as described above, for 15 min. Cells were washed once then chased with DMEM-10.

### Immunoprecipitation

Cells were harvested by scraping, pelleted by centrifugation at 5000 × g for 5 min, and then washed once with PBS and pelleted again. The pellet was resuspended in 200 μl lysis buffer (100 mM Tris-HCl, pH 8.0; 100 mM NaCl; 1.0% Triton-X 100) containing Complete, Mini protease inhibitors at a 1× concentration (Roche, Indianapolis, IN), and incubated on ice for 10 min. After removing nuclei by centrifugation, lysates were pre-cleared by incubation with Protein G-Sepharose (Amersham Pharmacia Biotech, Piscataway, NJ) for 30 min. Lysates derived from a VLP detection assay were frozen at -80°C until immunoprecipitation. Typically 1–2 μl of appropriate antiserum was used for each sample and antisera and lysates were incubated at 4°C overnight, followed by addition of Protein G-Sepharose for 45 min. Protein G beads were washed twice with lysis buffer followed by one wash with lysis buffer containing 0.1% sodium deoxycholate and 0.1% SDS. Proteins were separated either by SDS-polyacrylamide gel electrophoresis (SDS-PAGE) on a 10% polyacrylamide gel, or by a NuPAGE Novex 4–12% Bis-Tris gel (Invitrogen, Gaithersburg, MD) and visualized by autoradiography.

### VLP assay

293T cells were transfected, and Vero cells were infected with recombinant MVAs, as described above. At 20–24 h p.t. or 48 h p.i., the cell culture medium was removed, clarified, and then centrifuged through a cushion of 10% sucrose (w/vol) in NTE (100 mM NaCl; 10 mM Tris-HCl, pH 7.5; 1 mM EDTA) at 200,000 × g for 2 h at 4°C. The resulting pellet was re-suspended in 4 ml NTE and 1.3 ml 80% sucrose. A discontinuous sucrose gradient was formed by overlaying with 1.8 ml 50% followed by 0.6 ml 10% sucrose in NTE. The gradient was centrifuged at 200,000 × g for 16 h at 4°C in a SW50.1 rotor. Typically, two 0.7 ml fractions were removed from the top of the gradient, diluted with 2× lysis buffer, and analyzed by immunoprecipitation. The two fractions were combined during the first wash after Protein G-Sepharose addition. Proteins from 1/20 of cell lysates from MVA infections, and half of cell lysates from transfected cells, were used for immunoprecipitation. For samples derived from transfection, 1/3 of the sample was loaded on gels (equivalent to 1/6 total lysate). Release efficiency was quantified by performing densitometry analysis on scanned film images using AlphaEaseFC software (Alpha Innotech Corporation, San Leandro, CA). Percent release was calculated as the fraction of protein derived from the supernatant divided by total protein detected (total lysate + supernatant).

### Equilibrium centrifugation

Clarified culture supernatants of MVA-infected Vero cells were centrifuged at 200,000 × g for 2 h at 4°C. The resulting pellet was resuspended in 200 μl NTE and added to the top of a continuous gradient of 5–45% sucrose. Clarified supernatants from transfected cells were added directly to the gradient. The gradient was centrifuged in an SW40 Ti rotor at 200,000 × g for 16 h at 4°C. Fractions were collected from the bottom and 50 μl of each fraction was set aside for density measurement. The remainder of each fraction was combined with 4× lysis buffer (pH 7.5) and immunoprecipitated as described. Density was determined based on refractive index as measured with a refractometer. Culture supernatants of NiV-infected Vero cells were clarified at 20,000 × g for 10 min at room temperature and 100 μl of clarified supernatant containing approximately 10^6 ^TCID_50 _was added to the top of a continuous gradient of 5–45% sucrose. The gradient was centrifuged in an SW41 Ti rotor at 200,000 × g for 16 h at 4°C. Fractions were collected from the bottom and 50 μl of each fraction was set aside for density measurement. A portion (140 μl) of each fraction was extracted using the QIAamp Viral RNA Mini kit (Qiagen GmbH, Hilden) and extracted RNA was analyzed by quantitative real-time PCR as previously described [74]. Threshold cycle (Ct) values were used as a proxy for virus concentration and density was determined based on refractive index as measured with a refractometer.

### Electron microscopy

VLPs released from transfected 293T cells into the culture supernatant were prepared as described above except the top 1.4 ml of the flotation gradient was mixed with 3 ml of PBS and centrifuged for an additional 2 h after which the pellet was resuspended in 60 μl PBS. VLPs were adsorbed onto carbon-coated parlodion copper grids and immunolabeled using either polyclonal HeV G-specific mouse antiserum or anti-NiV M monoclonal antibody F45G4, followed by secondary antibody conjugated to 12 nm gold beads. For detection of M, immuno-labeling was done in the presence of 0.05% saponin. Samples were negatively stained with 1% uranyl acetate and examined with a Hitachi 7600 transmission electron microscope operated at 80 kV.

## Competing interests

The author(s) declare that they have no competing interests.

## Authors' contributions

JRP conceived and contributed to the development of the NiV VLP system, designed and constructed all expression constructs and carried out all recombinant expression and analysis assays, interpreted data, and wrote the first draft of the manuscript. GC developed and carried out all live virus infections and density analysis and interpreted data and edited and corrected the manuscript. LFW edited and corrected the manuscript. BTE provided expertise for conducting the live virus infection experiments, financial support, edited and corrected the manuscript. CCB conceived and contributed to the development of the NiV VLP system, provided overall supervision and financial support and prepared the final versions of the manuscript.
